# Toxicity and cosmetic outcome of hypofractionated whole-breast radiotherapy: predictive clinical and dosimetric factors

**DOI:** 10.1186/1748-717X-9-97

**Published:** 2014-04-24

**Authors:** Patrizia Ciammella, Ala Podgornii, Maria Galeandro, Renato Micera, Dafne Ramundo, Tamara Palmieri, Elisabetta Cagni, Cinzia Iotti

**Affiliations:** 1 Radiation Oncology Unit, Department of Advanced Technology, Arcispedale Santa Maria Nuova, Istituto di Ricovero e Cura a Carattere Scientifico, Viale Risorgimento 80, 42123 Reggio Emilia Italy; 2 Medical Physic Department, Arcispedale Santa Maria Nuova, Istituto di Ricovero e Cura a Carattere Scientifico, Reggio Emilia, Italy

## Abstract

**Purpose:**

The objective of this study is to evaluate toxicity and cosmetic outcome in breast cancer patients treated with adjuvant hypo fractionated radiotherapy to the whole breast, and to identify the risk factors for toxicity.

**Methods and materials:**

Two hundred twelve women with early breast cancer underwent conserving surgery were enrolled in the study. The patients received 40.05 Gy in 15 daily fractions, 2.67 Gy per fraction. The boost to the tumor bed was administered with a total dose of 9 Gy in 3 consecutive fractions in 55 women. Physician-rated acute and late toxicity and cosmetic outcome (both subjective and objective) were prospectively assessed during and after radiotherapy.

**Results:**

In our population study the mean age was 63 with the 17% (36 pts) of the women younger than 50 years.

The median follow-up was 34 months. By the end of RT, 35 patients out of 212 (16%) no acute toxicity, according to the RTOG criteria, while 145 (68%) and 31 patients (15%) developed grade 1 and grade 2 acute skin toxicity, respectively.

Late skin toxicity evaluation was available for all 212 patients with a minimum follow up of 8 months. The distribution of toxicity was: 39 pts (18%) with grade 1 and 2 pts (1%) with grade 2. No worse late skin toxicity was observed.

Late subcutaneous grade 0-1 toxicity was recorded in 208 patients (98%) and grade 2 toxicity in 3 patients (2%), while grade 3 was observed in 1 patient only. At last follow up, a subjective and objective good or excellent cosmetic outcome was reported in 93% and 92% of the women, respectively. At univariate and multivariate analysis, the late skin toxicity was correlated with the additional boost delivery (p=0.007 and p=0.023). Regarding the late subcutaneous tissue, a correlation with diabetes was found (p=0.0283).

**Conclusion:**

These results confirm the feasibility and safety of the hypofractionated radiotherapy in patients with early breast cancer. In our population the boost administration was resulted to be a significant adverse prognostic factor for acute and late toxicity. Long-term follow up is need to confirm this finding.

## Introduction

The radiation therapy represents the standard adjuvant treatment for the early-stage breast cancer after breast conserving surgery (BCS), in order to maximize local control and overall survival [1]. Adjuvant radiotherapy has been shown to improve local control and overall survival, with a 70% reduction in the risk of recurrence [2,3] and a 9-12% reduction in the risk of death [4-6]. The most widely used fractionation regimen is 1.8 to 2-Gy daily fractions for a total dose of 45 to 50 Gy to the whole breast over 5 weeks with or without a boost to the surgical bed.

Although there has been concern that the whole-breast radiotherapy using daily dose > 2 Gy/fraction might increase late toxicity and impair cosmesis in BCS patients [7], over the last years, there has been renewed interest in hypofractionated whole breast irradiation (HF-WBI), defined as a larger daily dose delivered often over a shorter time period. HF-WBI is associated with reduction in the length of treatment by 2-3 weeks compared to conventional schedules that require 6-7 weeks. This approach have important practical advantages and biological implications. Their convenience, also in terms of cost savings to the patient and the health care provider, may facilitate patient acceptance and compliance with radiotherapy. Large multicenter randomized trials with 5- to 10-year follow-up data have shown efficacy and safety in terms of local control and cosmetic outcome [8,9], however, only few studies have investigated cosmesis [10-12] and quality of life [11]. The first randomised trial was conducted in Canada and has tested 42.5 Gy in 16 fractions against 50 Gy in 25 fractions, resulting in equivalent local control and breast cosmesis [13]. The two most recent randomized studies, conducted in UK (START Trials), have demonstrated that the hypofractionation offers a favourable rates of late effects and loco-regional tumor control [8,13]. Because of the greater risk of worse fibrosis and skin toxicity, several studies hypo fractionation excluded the large-breasted women [8]; some other trials included these patients but without providing clear information about the impact of breast volume on toxicity and cosmesis [10,13].

The aim of the present prospective study is to assess the acute and late toxicity and the cosmetic outcome of a postoperative HF-WBI for early breast cancer, and to analyze their correlation with clinical and dosimetric characteristics.

## Methods and materials

### Characteristics of patients and data collection

From January 2009 and December 2012, two hundred twelve women with early breast cancer were recruited in this mono-institutional prospective trial of HF-WBI. The inclusion criteria were: age ≥ 18 years, histological proven unilateral early-breast cancer, prior conservative surgery (lumpectomy or quadrantectomy), pathological stage pT1-pT2, pN0-1 according to American Joint Committee-Union Internationale Contre le Cancer staging system (AJCC-UICC, 6^th^ edition), negative surgical margins. All patients provided an informed consent. Patients previously irradiated on the controlateral breast, with synchronous bilateral breast cancer, with > 4 positive lymph nodes, connective tissue disorders were excluded from the present study.

The diabetes mellitus and the chemotherapy were not considered factors of exclusion and indeed were analyzed as independent factors for toxicity. Our Institutional Ethic Committee approved this study. Adjuvant systemic therapy was prescribed after multidisciplinary evaluation and was mostly based on the most recent San Gallen Expert Meeting Consensus.

### Radiation treatment

The planning CT scan (5 mm slice thickness) from the level of the larynx to the upper abdomen were obtained in the supine position using “wing-board” or other personalized immobilization device with both arms raised above the head. The Clinical Target Volume (CTV) included whole breast tissue and was expanded by 5 mm to create the Planning Target Volume (PTV). Organs at risk (OARs), lungs and heart, were contoured.

A three dimensional conformal radiotherapy, planned with Eclipse Varian, was calculated both for whole breast and boost irradiation. Whole breast was treated to a total dose of 40.05 Gy in 15 consecutive daily fractions, 2.67 Gy per fraction. An additional dose of 9 Gy in three consecutive fractions was delivered in patients with risk factors for local relapse (age < 50 years or close surgical margins). The dose was prescribed to the ICRU reference point, according with the following constraints: 95% and 90% of the prescribed dose to the 95% and the 90% of the PTV respectively. For each patient, dose-volume histograms (DVHs) for the target and OARs were obtained.

### Follow-up, toxicity and cosmesis assessment

All patients underwent clinical examination before irradiation, weekly during the treatment course and every three months for the first year and then every six months. Surveillance for disease recurrence included a clinical examination at every time point, and mammography once a year. Acute skin toxicity was assessed during and at the completion of RT and after 3 months; late effects and cosmetic outcome were evaluated at each clinical visit.

Late skin and subcutaneous toxicity were evaluated in accordance with the RTOG grading schema [14,15]. For all patients the cosmetic outcome was evaluated by patient (subjective score) and physician (objective score). The cosmetic assessment was performed by the physician using the contralateral, untreated breast as the reference. The cosmetic outcome was scored using the Harvard scale [16]. An excellent cosmetic result score was assigned when the treated breast looked like essentially the same as the contra lateral one (as it relates to radiation effects). A good cosmetic score was assigned for minimal but identifiable radiation effects of the treated breast. A fair score meant that significant radiation effects were readily observable. A poor score was used for severe sequelae of breast tissue secondary to radiation effects.

A patient-based cosmetic evaluation was done according to the standard European Organization for Research and Treatment of Cancer (EORTC) Breast Cancer Rating System for Cosmetic Results of Breast Conserving Treatment. In brief, they were asked to compare their treated breast with the untreated breast and grade the following items: breast size and shape, location and shape of areola/nipple, skin color, breast edema, appearance of surgical scar, telangiectasia, and global cosmetic result. Items were graded on the following four-point scale: no difference or excellent, small difference or good; moderate difference or fair, and large difference or poor.

The patients (age, comorbidities, body mass index) and medical treatments (neoadjuvant/adjuvant chemotherapy, hormonal deprivation, other concomitant drugs) characteristics were analyzed and correlated with toxicity and cosmetic outcome. Furthermore, we analyzed the impact of the CTV volume (representing the breast size), and the dosimetric data, with special focus on the maximum dose and dose in homogeneity (defined as the absolute volumes of breast tissues exposed to ≥100%, ≥104% and ≥107% of the prescribed).

### Statistical analysis

The χ2 and Mann–Whitney tests were used to compare acute skin toxicity and late skin and subcutaneous toxicity between different sample groups and to analyze associations between toxicity and cosmetic outcome with clinical characteristics. Multivariate analysis to independently predict the risk of skin and subcutaneous toxicity development was performed using binary logistic regression. The absolute breast volumes receiving >100%, >104% and >107% of the prescribed doses (40.05 Gy and 49.05 Gy for patient receiving an adding boost respectively) were summarized and correlated with the subcutaneous toxicity. For each dose level, medians were used to split the distribution into two groups for the analysis: <median and <median. Multiple logistic regression was used to test the association between breast volume receiving different levels of prescribed dose and late subcutaneous toxicity for whole breast and boost treatment schedules.

Statistical significance was assumed at p < 0.05; data were processed using the R Package Version 2.15.

## Results

The mean age was 63 (range 39-88 yrs) with the 17% of the women younger than 50 years. Twenty three (11%) and twenty five (12%) patients were affected by diabetes mellitus and hypertension, respectively. 15% had tumors with a diameter ≥ 2 cm; 12% had hormone-receptor–negative disease and 26% had poorly differentiated disease. All patients received prior breast conserving surgery (no tumorectomy). In this study, 24 patients had close margins (within 2 mm from the surgical margin but not tumor on ink) at final pathological examination. Thirty four patients underwent level I/II axillary lymph-nodes dissection and 165 sentinel node biopsy alone. The most common site was supero-externe quadrant (103, 48%) followed by supero-central (38, 18%) and lower outer quadrant (17, 8%). One hundred fourthy six patients (64%) had T1 tumors 32 patients (15%) had T2 tumors, 41 (19%) had *in situ* tumor. Invasive ductal carcinoma was the commonest pathological type (152, 72%) while invasive lobular carcinoma was found in only 17 patients (8%). Neo-adjuvant chemotherapy was administered in 10 patients; 34 patients received adjuvant chemotherapy and 170 hormone therapy. The chemotherapy regimens were antracycline-based (± taxanes). Eighteen patients (8%) underwent trastuzumab therapy.

Patients characteristics and details of treatments are shown in Tables 1 and 2.

**Table 1:** Patient and tumor characteristics

**Characteristics**	**Total patients**
	**N (%)**
**No.**	212
***Mean age (range)***	63 (39-88)
***Diabetes Mellitus***	23 (11%)
***Hypertension***	25 (12%)
***Breast side***	
Right	110 (48%)
Left	102 (52%)
***Breast Quadrant***	
SE	103 (48%)
SC	38 (18%)
II	12 (6%)
IE	17 (8%)
IC	11 (5%)
CE	8 (4%)
SI	23 (11%)
***Histological type***	
IDC	152 (72%)
ILC	17 (8%)
Intraductal	41 (19%)
Other	1 (1%)
***T stage***	
T0	3 (2%)
T is	41 (19%)
T 1a	11 (5%)
T 1b	43 (20%)
T 1c	82 (39%)
T 2	32 (15%)
***N stage***	
Nx	13 (6%)
N0	165 (78%)
N1	34 (16%)
***Grading***	
G1	32 (15%)
G2	118 (56%)
G3	56 (26%)
NA	6 (3%)
***Surgical margins***	
Negative	186 (82%)
Positive	5 (2%)
Close (< 2 mm)	19 (9%)
NA	1 (1%)
***Chemotherapy***	
Yes	44 (21%)
(Neoadjuvant)	10
(Adjuvant)	34
No	167 (79%)
***Hormone therapy***	
Yes	159 (75%)
No	42 (20%)
NA	11 (5%)
***Trastuzumab***	
Yes	18 (8%)
No	194 (92%)

IDC – infiltrating ductal carcinoma; ILC – infiltrating lobular carcinoma; NA - not applicable; SE – supero-interne; II – infero-interne; SE – supero-externe; IE – infero-externe; SC – supero-central; IC – infero-central; SI – supero-interne; CE - central.

**Table 2:** Treatment and dosimetric characteristics

***Breast Volume***	
Average cc	813.8
(Range)	(89.6 - 1892.1)
***Boost Total patients***	55 (26%)
***Boost Volume***	
Average cc	138.75
(Range)	(23.07 - 230.02)
***PTV (Gy)***	
Mean dose:	41.15
Median dose:	41.09
***Mean Maximum Dose (Gy)***	43.65
***Breast volume riceiving ≥ 100% dose (cc)***	
(Range)	79.7 - 1520.7
***Breast volume receiving ≥ 104% dose (cc)***	
(Range)	13.1 - 905
***Breast volume receiving ≥ 107% dose (cc)***	
(Range)	0 - 549.8

Median time from surgery to RT was 65 days with overall median radiation treatment duration of 22 days. No patient interrupted the treatment.

The median follow-up was 34 months (range 8-44 months). At last follow up all patients are alive without local recurrence.

The median breast volume was 760.64 cc (range 44.77-1892.1 cc). The median boost volume was 143.33 cc (range 23.07-230.02 cc). The median breast volume for each isodose group >100%, >104% and >107% were 562 cc (range 79.7-1520.7 cc), 216.6 cc (range 13.1-905 cc) and 13.6 cc (range 0-549.8 cc), respectively.

### Acute toxicity

**Figure 1:**
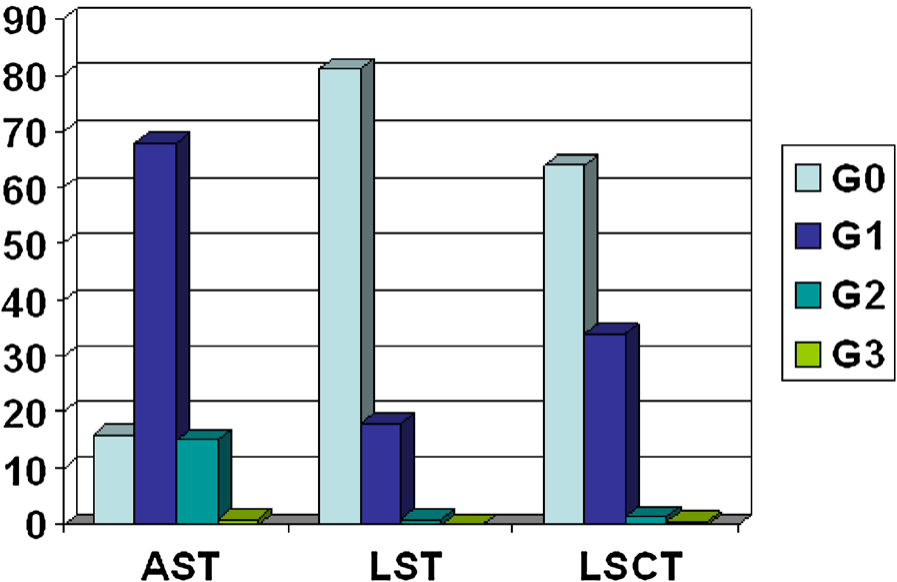
Distribution of acute skin toxicity (AST), late skin toxicity (LST) and late subcutaneous toxicity (LSCT).

For simplicity, patients were considered to have mild skin reaction for those with G1, moderate skin reaction for those with G2, and severe skin toxicity for those with G3-G4 reaction. The overall frequency of acute toxicity was reported in Table 3. Mild acute skin toxicity was observed in 168 patients (79%); 12% of patients developed moderated skin toxicity and only one patient, affected by diabetes mellitus and obesity, experienced a grade 3 reaction. The remaining 39 patients (18%) showed no acute toxicity. Neither skin ulceration nor soft tissue necrosis (grade 4 toxicity) was observed. Distribution of Acute Skin Toxicity (AST), Late Skin Toxicity (LST) and Late Subcutaneous Toxicity (LSCT) according to RTOG scale was shown in Figure 1.

**Table 3:** Frequency of any grade of acute skin toxicity

**RTOG**	**Total patients**
**Toxicity grade**	**N (%)**
G0	35 (16%)
G1	144 (68%)
G2	32 (15%)
G3	1 (1%)

Median delay between RT initiating and first skin reaction was 12 days (range: 5-40 days). Median dose to first skin reaction was 29.37 Gy (range 13.35-40.05 Gy). No treatment disruption was necessary.

Patient and treatment related factors have been evaluated in a univariate analysis (Table 4). At Mann-Whitney test only the boost administration was found to be statistically significant as concerns the occurrence of acute skin reaction (p = 0.001156). In our analysis other clinical factors such as age, smoking, hypertension and chemotherapy were not correlated with the development of acute skin reaction.

**Table 4:** Univariate and multivariate analysis: predictive factors for acute skin radiation – induced toxicity

**Variables**	**Univariate analysis**	**Multivariate analysis**
	***p*** **value**	***p*** **value [OR]**
Chemotherapy	0.443	0.752
Hypertension	0.805	0.782
Age	0.200	0.373
Breast volume	0.696	= 0.01504 [OR 1.00612 (0.99659 – 1.01588)]
Diabetes	0.149	0.568
Surgical deficits	0.107	= 0.00383 [OR 3.4577 (1.4914 – 8.0161)]
Boost administration	= 0.0001156	= 0.00543 [OR 0.9909 (0.98363 – 0.9982)]
V > 100%	-	0.608
V > 104%	-	0.323
V > 107%	-	0.279
Boost V > 100%	-	0.997
Boost V > 104%	-	0.997
Boost V > 107%	-	0.546

A significant correlation by Binary logistic regression test between breast volume [p = 0.01504, Odds Ratio, OR 2.4738 (0.9834-6.2230)], boost administration [p = 0.05431, OR 0.9909 (0.98363-0.9982)] and surgical outcomes [p = 0.00383, OR 3.4577 (1.4914-8.0161)] was found for the occurrence of acute toxicity. Analyzing separately the “no boost” group, the only and unexpected variable retaining significance was the surgical deficits [p = 0.00061, OR 7.665326 (2.405551-24.42568)].

Results from the multivariate analysis suggested that after adjusting for breast size, boost administration and surgical deficits, there was no evidence that the risk of acute skin effects of radiotherapy was associated with dose inhomogeneity.

### Late toxicity

Late toxicity was assessed at 6 months from the end of radiotherapy and thereafter. The frequencies of late skin and subcutaneous toxicity was reported in Tables 5 and 6.

**Table 5:** Frequency of any grade of late skin toxicity

**RTOG**	**Total patients**
**Toxicity grade**	**N (%)**
G0	171 (81%)
G1	39 (18%)
G2	2 (1%)
G3	0 (0%)

**Table 6:** Frequency of any grade of late subcutaneous toxicity

**RTOG**	**Total patients**
**Toxicity grade**	**N (%)**
G0	136 (64%)
G1	72 (34%)
G2	3 (1.5%)
G3	1 (0.5%)

Late G1 skin toxicity, scored with RTOG scale, was observed in 39 patients (18%); only 2 patients showed G2 late skin reactions. No late skin toxicity > grade 2 was observed. At univariate analysis, as summarized in Table 7 only the boost administration was related to late skin toxicity (p = 0.0007174). This correlation was also confirmed by the multivariate analysis [p = 0.019, OR 3.056 (1.280-7.297)]. There was a higher but not statistically significant incidence of late skin toxicity in the patients who developed early skin reaction (p = 0.2183).

**Table 7:** Univariate and multivariate analysis predictive factors for late radiation induced skin toxicity

**Variables**	**Univariate analysis**	**Multivariate analysis**
	***p *** **value**	***p *** **value [OR]**
Chemotherapy	0.118	0.232
Hypertension	0.949	0.898
Age	0.603	0.087
Breast volume	0.620	0.692
Diabetes	0.196	0.139
Surgical deficits	0.323	0.890
Boost administration	= 0.007174	= 0.0119 [OR 3.056 (1.280 - 7.297)]
V > 100%	-	0.642
V > 104%	-	0.466
V > 107%	-	0.908
Boost V > 100%	-	0.981
Boost V > 104%	-	0.684
Boost V > 107%	-	0.615

Late subcutaneous G0-G1 toxicity was recorded in 98% of patients; G2 toxicity was observed in 2% of patients and only in 1 patient showed G3 toxicity. At Mann-Whitney test only the diabetes was found to be statistically significant as concerns the occurrence of late subcutaneous toxicity (p = 0.0283), while at multivariate analysis the only and unexpected variable retaining significance was the chemotherapy administration [p = 0.0184, OR 2.5923 (1.1745-5.7217)] (Table 8). With regard to the dosimetric factors, both the V104 [p = 0.00864, OR 0.07604728 (0.01122575-0.5151717)] and V107 [p = 0.02045, OR 6.268894 (1.338293-29.36504)] were found to be related to the chronic subcutaneous toxicity. Analyzing the “no boost” group, the only variable correlated with late subcutaneous toxicity was the diabetes [p = 0.00350, OR 3.514025 (1.092603-11.30179)].

**Table 8:** Univariate and multivariate analysis predictive factors for late radiation induced subcutaneous toxicity

**Variables**	**Univariate analysis**	**Multivariate analysis**
	***p *** **value**	***p *** **value [OR]**
Chemotherapy	0.118	= 0.0184 [OR 2.5923 (1.1745 – 5.7217)]
Hypertension	0.705	0.731
Age	0.956	0.223
Breast volume	0.694	0.483
Diabetes	= 0.0283	0.055
Surgical deficits	0.854	0.499
Boost administration	0.5157	0.298
V > 100%	-	0.745
V > 104%	-	= 0.00864 [OR 0.07605 (0.01122 – 0.51517)]
V > 107%	-	= 0.02045 [OR 6.26889 (1.33829 – 29.36504)]
Boost V > 100%	-	0.728
Boost V > 104%	-	0.099
Boost V > 107%	-	0.585

### Cosmetic outcome

**Figure 2:**
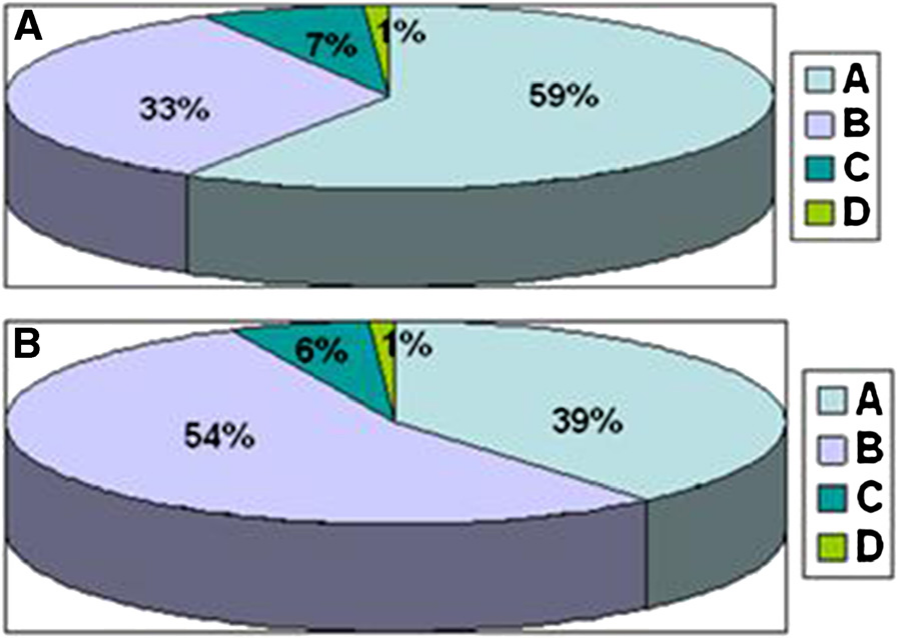
Distribution of (A) objective (OCO) and (B) subjective cosmetic outcome (SCO): (A) excellent cosmeses, (B) good cosmeses, (C) fair cosmeses and (D) poor cosmeses.

Cosmetic results were assessed and scored at the end of RT and then every 6 months. The distribution of objective and subjective cosmetic outcome was showed in Figure 2.

At last follow up, 197 women (93%) declared a subjective good or excellent cosmetic outcome; the objective score, recorded by physician, was good or excellent in 196 (92%) patients. Fifteen patients (7%) got unsatisfactory subjective cosmetic results (fair in 12 cases and poor in 3). The physician reported a poor outcome only in two patients and fair results in 14.

**Figure 3:**
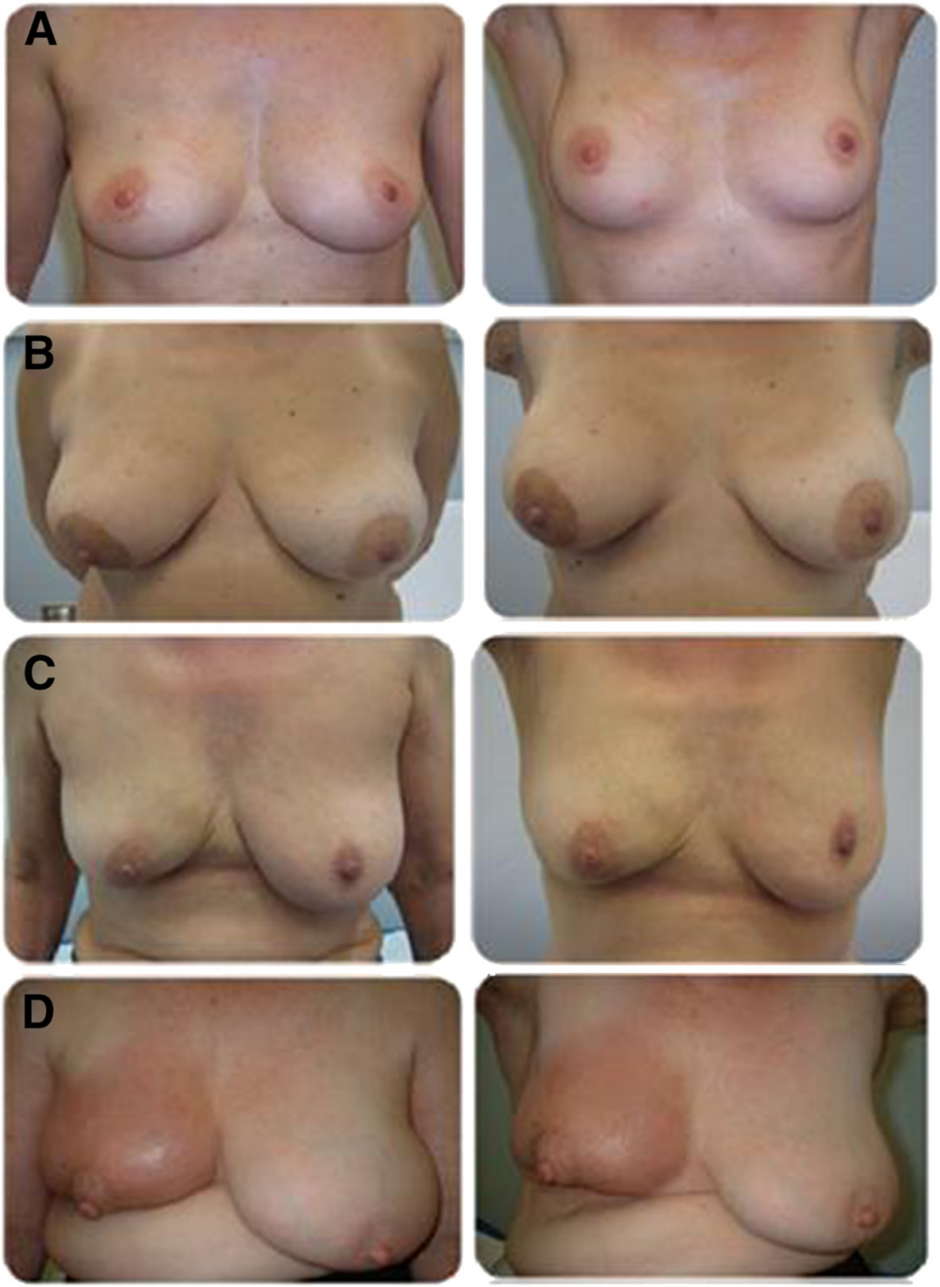
Examples of objective cosmetic results (arms up and arms down): (A) excellent cosmeses, (B) good cosmeses, (C) fair cosmeses and (D) poor cosmeses.

Figure 3 shows an examples of excellent (A), good (B), fair (C) and poor (D) cosmetic results. The univariate analysis of the subjective results showed that diabetes was correlated with a poor outcome (p = 0.02377); this result was confirmed also with the multivariate analysis [p = 0.0596, OR 0.2767931 (0.07273-1.053304)]. With regard to cosmetic outcomes assessed by physicians, the binary logistic regression showed that age [p = 0.003141, OR 0.8985 (0.8370-0.9647)], chemotherapy administration [p = 0.0409, OR 0.108474 (0.02440211-0.482173)] and breast volume [p = 0.0207042, OR 1.00619 (0.9965905-1.015882)] were correlated with fair-poor results.

## Discussion

The delivery of daily doses higher than 1.8-2 Gy with hypofractionated schedule is a widespread option to perform whole breast irradiation after breast-conserving surgery for early-breast cancer [17]. The HF-WBI has been used in several institutions for decades and tested in randomized trials [18,19]. There are three randomized trials performed in the last years that have compared hypofractionation with conventional radiotherapy for whole breast irradiation. In the Canadian trial 1,234 women with early-breast cancer were randomized after breast conserving surgery to accelerated HF-WBI (42.5 Gy/16 fx) or standard course (50 Gy/25 fx). This study demonstrated with a median follow up of 12 years a comparable results between two groups of patients in terms of local control and adverse events [20]. The UK standardization of breast radiotherapy (START) Trial A enrolled 2.236 patients randomized to conventional radiation therapy versus two different schedules of hypofractionation (41.6 or 39 Gy in 13 fractions) [13]. In the START B trial, 2215 women with breast cancer were randomized after breast conserving surgery or mastectomy to standard whole breast irradiation (50 Gy/25 fx) or accelerated HF-WBI (40 Gy/15 fx) [8]. Both trials showed similar outcomes with respect to local recurrence and adverse cosmesis for patients treated with hypofractionated regimens compared with standard treatment. Despite the uniformity of the results, there are several differences in patient selection, length of follow-up and use of systemic therapy and radiation boost among the three trials. In the Canadian study no patient received boost irradiation and only 10.9% received adjuvant systemic therapy. Furthermore, women with large breast were excluded because of an increase in adverse cosmesis observed when such patients were treated with standard fractionation [21,22]. In contrast, in the START A and B Trials no exclusion criteria based on breast size were applied; 36% of patients received adjuvant systemic therapy and, although its use was not standardized, most patients received a radiotherapy boost. The late toxicity and adverse cosmetic results were measured and reported differently in these three trials, as well. The Canadian trial used the Radiation Oncology Group/European Organization for Research and Treatment of Cancer (RTOG/EORTC) late scoring schema for skin and subcutaneous tissues, while the cosmetic outcome was assessed by a clinical trial nurse at baseline and 3, 5 and 10 years using the EORTC cosmetic rating system. All these differences, as emphasized in a recent review [23], have precluded the widespread acceptance of hypofractionated approach in United States, so much so that the American Society of Therapeutic Radiology and Oncology (ASTRO) guidelines reported that the HF-WBI was appropriate in patients of 50 years or older at diagnosis, with pathological stage T1-T2 N0 disease treated with breast conserving surgery, without chemotherapy and with a dose inhomogeneity on radiation plan <7%. A consensus on the applicability of HF-WBI to young patients and those underwent chemotherapy and/or boost was unable to be reached, based on the lack of mature clinical data on these patient subsets.

The most common change in breast appearance after radiotherapy is shrinkage, edema, retraction and teleangectasia. The tissue induration persistent many years after radiotherapy usually is due to an underlying fibrosis, but in the early years the fat necrosis and breast edema can contribute to induration scores. The adverse effects will appear for as long as patients are alive and the median follow-up times of the hypofractionated trials varied from 5.1 and 9.7 years. The critical question, as emphasized by Yarnold and colleagues [24], is whether the fractionation sensitivity of responses developing at the time of reporting are representative of those developing over entire life span of a patient. Curran et al. showed that cosmesis after breast conserving therapy was worse if patients were followed up much longer than 5 years [25]. On the contrary, the UK Royal Marsden Hospital/Gloucestershire Oncology Centre (RMH/GOC) trial [26] did not show a difference between 5-year and 10-year late adverse. On the basis of these considerations and uncertainties, nowadays may be unjustified to consider follow-up a factor limiting the interpretation of current hypofractionation trials [27]. Similarly to what reported in other literature studies, our study showed an overall low risk of late skin and subcutaneous toxicity in patients treated with HF-WBI after breast-conserving surgery with a median follow up of 34 months. In the present study patients with less than 50 years were enrolled. In literature, some authors used age as a selection criteria [12] as well as breast size or large chest wall separation. In addition, the published trials of HF-WBI consisted of mostly lower-risk patients, therefore the applicability and safety of hypofractionation in women treated with adjuvant chemotherapy is not well known. In the past, the worsening effect of chemotherapy on long-term fibrosis and cosmetic outcome was reported [28,29]. The impact of the modern anthracycline- and taxane-based regimens in patients treated with HF-WBI is unknown. In our study patients treated with adjuvant chemotherapy (mainly anthracycline-based) showed a significant increase of late subcutaneous toxicity (p = 0.0184) and a poor objective cosmetic outcome (p = 0.0409) at multivariate analysis. Anyway, the rate of high grade scores remained low.

In regard to the use of a tumor bed boost, the question “when tumor bed boost is recommended and what is the optimal method of delivering it with hypofractionated irradiation” is still an unsolved issue. In prospective randomized trials the use of a tumor bed boost after whole breast irradiation reduced the risk of local recurrence even in patients with negative margins [30,31]. Besides, an international survey showed that 85% and 75% American and European physicians, respectively, would deliver a boost even with negative margins [32]. The prospective trials of HF-WBI or never used a boost or used it at the discretion of the treating physician or department policy. Recent phase I-II trials investigated the role of concomitant boost in HF-WBI showing the safety and short-term efficacy of this approach for early-breast cancer. Freedman et al. treated 75 women affected by early-stage breast cancer using intensity modulated radiation technique (IMRT) with a whole breast dose of 2.25 Gy per day for 20 fractions and with an incorporated tumor bed boost of 2.8 Gy per fraction for a total dose of 56 Gy [33]. All breast sizes and chemotherapy administration were permitted. No excess of acute skin toxicity was found. Another clinical study of HF-WBI (40.5 Gy in 15 fractions) and concomitant boost (+0.5 Gy per day for a total tumor bed dose of 48 Gy) was carried out on 91 patients with early breast cancer. The major acute skin toxicity was a reversible grade 1-2 dermatitis occurred in 67% of the patients; there were 2 cases of acute grade 3 toxicities and no late grade 3 effects. The authors reported a grade 1 and 2 late soft tissue fibrosis in 40% and 3% of patients, respectively [34]. Furthermore, with a median follow-up of 24 months Chadha et al. reported any significant negative effect of HF-WBI and concomitant boost on breast cosmesis [35]. So far, neither the optimal HF-WBI regimen to use when a boost is planned nor the optimal tumor bed boost dose fractionation have been determined. In our study we used the sequential tumor bed boost in patients ≤50 years or with close margins. A total dose of 9 Gy in three consecutive daily fractions with a non-coplanar 3D-CRT technique, was delivered. The bed tumor boost resulted significantly associated with an higher grade of acute and late skin toxicity (p = 0.05431 and p = 0.0119). Analyzing only the “boost” group, the multivariate analysis did not show any other clinical factors related to skin toxicity, as if to signify that the boost is the main factor involved. As concerns breast volume as a relevant factor related to skin toxicity contradictory data are available in the literature. Some authors reported a strict correlation between breast volume size and severity of acute effects, because large volumes are frequently associated with dose inhomogeneity and maximum doses higher than the 107% of prescribed dose [36,37]. In addition to acute toxicity, breast volume seems to increase the risk of late effects, too [37].

On the contrary, Corbin et al. reported that among obese and large breasted women, in their experience, there was no increase in acute skin toxicity [38]. One possible explanation for these discrepancies may be the different criteria used to define “large breast”. Vicini et al. [28] found that patients with breast volume > 1600 cc had more acute skin toxicity than those with smaller breast. Harsolia showed no grade 3 RTOG acute toxicity with breast volume <975 cc; patients with breast volumes > 1600 cc developed 59% G2 and 3% G3 RTOG skin reactions [29]. In this report, as in our study, the authors measured breast volume manually contouring of breast target volume. The average breast volume in our patients were smaller (813.8 cc, range 44.77-1892.1 cc) than reported in these studies but were substantially comparable to those recently reported by Tortorelli who showed a significant correlation between breast volume and acute skin toxicity at univariate analysis (p = 0.011) [39]. Our study confirmed a significant correlation between acute skin toxicity and breast volume (p = 0.01504). Also, it showed a significant correlation between breast size and objective cosmetic outcome (p = 0.0207).

In our experience, compliance with this treatment was excellent thanks to short treatment duration. The overall objective cosmetic outcome was generally good, with satisfactory cosmetic results in nearly 90% of patients. A thorough patients’ judgment of their own cosmetic outcomes revealed similar findings, and a very low rate of results rated as “poor”. This was expected as it has been already reported that patients tend to evaluate the esthetic outcomes more positively than health care providers.

As concerns the dosimetric inhomogeneities as a relevant factor related to acute and late toxicity a recent study suggested that the amount of volume receiving >107% was correlated with skin reaction [39] as pointed out by Chen and coll. who demonstrated that larger volume receiving >53.9 Gy was a significant predictor of radiation induced skin toxicity [40]. The recent UK FAST trial reported that, after adjusting for breast size and surgical deficits, there was no evidence that the risk of late adverse effects of HF-WBI was associated with dose inhomogeneity [41]. Conversely, our results suggest that dose inhomogeneities (V > 104% and V > 107%) have a significant impact on the occurrence of severe subcutaneous late reaction (p = 0.00864 and p = 0.02045, respectively) but we did not find any correlation between dosimetric parameters and both acute and late skin toxicity. Special focus should be given to dose inhomogeneity which do not correlate with skin reaction, because it may be related to other types of side effects which have not yet been investigated and reported in the aferomentioned randomized trials, such as late subcutaneous toxicity.

## Conclusions

The results of our study, according to the large randomized trials, confirmed that HF-WBI is safe. Our study showed an increase of late subcutaneous and skin effects in the patients who received additional boost. Anyway, the rate of severe toxicity (> grade 2) was low even in these patients. Then, the number of toxicity events is so low that no firm conclusion can be drawn from our data regarding the oncological safety of this procedure in patients needing of the additional boost.

## Competing interests

All authors disclose no actual or potential conflict of interest including any financial, personal or other relationships with other people or organizations that could inappropriately influence their work.

## Authors’ contributions

PC, MG, RM and CI participated in the design of the study. PC, AP, RM, DR, MG and TP carried out the data and participated in the data evaluation. PC, AP, EC and AB performed the statistical analysis. PC and AP drafted the manuscript. The definitive supervision of the paper was done by CI. All authors read and approved the final manuscript.

## References

[B1] Clarke M Collins R Darby S Davies C Elphinstone P Evans E Godwin J Gray R Hicks C James S MacKinnon E McGale P McHugh T Peto R Taylor C Wang Y for the Early Breast Cancer Trialists' Collaborative Group (EBCTCG) Effects of radiotherapy and of differences in the extent of surgery for early breast cancer on local recurrence and 15-year survival: an overview of the randomised trials Lancet 2005 366 2087 2106 16360786 10.1016/S0140-6736(05)67887-7

[B2] Cuzick J Rdaiotherapy for breast cancer J Natl Cancer Inst 2005 97 406 407 10.1093/jnci/dji086 15769997

[B3] Nielsen HM Overgaard M Grau C Jensen AR Overgaard J Study of failure pattern among high-risk berast cancer patients with or without postmastectomy radiotherapy in addition to adjuvant systemic therapy: long-term results from Danish Breast Cancer Cooperative Group DBCG 82 b and c randomized studies J Clin Oncol 2006 24 2268 2275 10.1200/JCO.2005.02.8738 16618947

[B4] Van de Steen J Soete G Storme G Adjuvant radiotherapy for breast cancer significantly improves overall survival: the missing link Radiother Oncol 2000 55 263 272 10.1016/S0167-8140(00)00204-8 10869741

[B5] Vinh-Hung V Verschraegen C The Breast Conserving Surgery Project. Breast conserving surgery with or without radiotherapy: pooled-analysis for risks of ispilateral breast tumor recurrence and mortality J Natl Cancer Inst 2004 96 115 121 10.1093/jnci/djh013 14734701

[B6] Taylor ME Haffty BG Rabinovich R Arthur DW Halberg FE Strom EA White JR Cobleigh MA Edge SB ACR appropriateness criteria on postmastectomy radiotherapy expert on radiation oncology-breast Int J Radiat Oncol Biol Phys 2009 73 997 1002 10.1016/j.ijrobp.2008.10.080 19251087

[B7] Thames HD Bnetzen SM Turesson I Ovrgaard M Van den Bogaert W Time-dose factors in radiotherapy: a review of the human data Radiother Oncol 1990 19 219 235 10.1016/0167-8140(90)90149-Q 2281152

[B8] Bentzen SM Agrawal RK Aird EG Bentzen SM Agrawal RK Aird EG Barrett JM Barrett-Lee PJ Bentzen SM Bliss JM Brown J Dewar JA Dobbs HJ Haviland JS Hoskin PJ Hopwood P Lawton PA Magee BJ Mills J Morgan DA Owen JR Simmons S Sumo G Sydenham MA Venables K Yarnold JR START Trialists' Group The UK Standardisation of Breast Radiotherapy (START) Trial B of radiotherapy hypofractionation for treatment of early breast cancer: a randomised trial Lancet 2008 371 1098 1107 18355913 10.1016/S0140-6736(08)60348-7 PMC2277488

[B9] Whelan TJ Kim DH Sussman J Clinical experience using hypofractionated radiation schedules in breast cancer Semin Radiat Oncol 2008 18 257 264 10.1016/j.semradonc.2008.04.008 18725113

[B10] Whelan T MacKenzie R Julian J Levine M Shelley W Grimard L Lada B Lukka H Perera F Fyles A Laukkanen E Gulavita S Benk V Szechtman B Randomized trial of breast irradiation schedules after lumpectomy for women with lymph node-negative breast cancer J Natl Cancer Inst 2002 94 15 1143 1150 10.1093/jnci/94.15.1143 12165639

[B11] Hopwood P Haviland JS Sumo G Mills J Bliss JM Yarnold JR Comparison of patient-reported breast, arm, and shoulder symptoms and body image after radiotherapy for early breast cancer: 5-year follow-up in the randomised Standardisation of Breast Radiotherapy (START) trials Lancet Oncol 2010 11 231 240 10.1016/S1470-2045(09)70382-1 20138809

[B12] Taher AN El-Baradie MM Essa H Zaki O Ezzat S Hypofractionation versus **conventional** fractionation radiotherapy after conservative treatment of breast cancer: early skin reactions and cosmetic results J Egypt Natl Canc Inst 2004 16 178 187 15959551

[B13] Bentzen SM Agrawal RK Aird EG Barrett JM Barrett-Lee PJ Bliss JM Brown J Dewar JA Dobbs HJ Haviland JS Hoskin PJ Hopwood P Lawton PA Magee BJ Mills J Morgan DA Owen JR Simmons S Sumo G Sydenham MA Venables K Yarnold JR START Trialists' Group The UK Standardisation of Breast Radiotherapy (START) Trial A of radiotherapy hypofractionation for treatment of early breast cancer: a randomised trial Lancet Oncol 2008 9 331 341 18356109 10.1016/S1470-2045(08)70077-9 PMC2323709

[B14] Cox JD Stetz J Pajak TF Toxicity criteria of the Radiation Therapy Oncology Group (RTOG) and the European Organization for Research and Treatment of Cancer (EORTC) Int J Radiat Oncol Biol Phys 1995 31 1341 1346 10.1016/0360-3016(95)00060-C 7713792

[B15] Rubin P Constine LS (RTOG Late Effects Working Group). Overview: late effects of normal tissues (LENT) scoring system Int J Radiat Oncol Biol Phys 1995 31 1041 1042 10.1016/0360-3016(95)00057-6 7713774

[B16] Trombetta M Julian TB Kim Y Werst ED Parda D The allegheny general modification of the Harvard Breast Cosmesis Scale for the retreated breast Oncology 2009 23 954 956 19947346

[B17] Coles CE Brunt AM Wheatley D Mukesh MB Yarnold JR Breast radiotherapy: less is more? Clin Oncol 2013 25 127 134 10.1016/j.clon.2012.10.013 23183306

[B18] Holloway CL Panet-Raymond V Olivotto I Hypofractionation should be the new “standard” for radiation therapy after breast conserving surgery Breast 2010 19 163 167 10.1016/j.breast.2010.03.002 20511064

[B19] Lievens Y Hypofractionated breast radiotherapy: financial and economic consequences Breast 2010 19 192 197 10.1016/j.breast.2010.03.003 20511069

[B20] Whelan TJ Pignol JP Levine MN Julian JA MacKenzie R Parpia S Shelley W Grimard L Bowen J Lukka H Perera F Fyles A Schneider K Gulavita S Freeman C Long-term results of hypofractionated radiation therapy for breast cancer N Engl J Med 2010 362 513 520 10.1056/NEJMoa0906260 20147717

[B21] Mayles WP Bliss JM A'Hern RP Owen JR Regan J Broad B Yarnold J The influence of breast size on late radiation effects and association with radiotherapy dose inhomogeneity Radiother Oncol 1994 33 106 112 10.1016/0167-8140(94)90063-9 7708953

[B22] Olivotto IA Weir LM Kim-Sing C Bajdik CD Trevisan CH Doll CM Lam WY Basco VE Jackson SM Late cosmetic results of short fractionation for breast conservation Radiother Oncol 1996 41 7 13 10.1016/S0167-8140(96)91824-1 8961362

[B23] Jonathan TY Alice Y Radiation therapy in the management of breast cancer Surg Clin N Am 2013 93 455 471 10.1016/j.suc.2013.01.002 23464696

[B24] Yarnold J Bentzen SM Coles C Haviland J Hypofractionated whole-breast radiotherapy for women with early breast cancer: myths and realities Int J Radiat Oncol Biol Phys 2011 79 1 9 10.1016/j.ijrobp.2010.08.035 20950960

[B25] Curran D van Dongen JP Aaronson NK Kiebert G Fentiman IS Mignolet F Bartelink H Quality of life of early-stage breast cancer patients treated with radical mastectomy or breast-conserving procedures: results of EORTC Trial 10801 Eur J Cancer 1998 34 307 314 10.1016/S0959-8049(97)00312-2 9640214

[B26] Yarnold J Ashton A Bliss J Homewood J Harper C Hanson J Haviland J Bentzen S Owen R Fractionation sensitivity and dose response of late adverse effects in the breast after radiotherapy for early breast cancer: long-term results of a randomised trial Radiother Oncol 2005 75 9 17 10.1016/j.radonc.2005.01.005 15878095

[B27] Bartelink H Arriagada R Hypofractionation in radiotherapy for breast cancer Lancet 2008 371 1050 1052 10.1016/S0140-6736(08)60349-9 18355914

[B28] Vicini FA Sharpe M Kestin L Martinez A Mitchell CK Wallace MF Matter R Wong J Optimizing breast cancer treatment efficacy with intensity-modulated radiotherapy Int J Radiat Oncol Biol Phys 2002 54 5 1336 1344 10.1016/S0360-3016(02)03746-X 12459355

[B29] Harsolia A Kestin L Grills I Wallace M Jolly S Jones C Lala M Martinez A Schell S Vicini FA Intensity-modulated radiotherapy results in significant decrease in clinical toxicities compared with conventional wedge-based breast radiotherapy In J Radiat Oncol Biol Phys 2007 68 5 1375 1380 10.1016/j.ijrobp.2007.02.044 17544598

[B30] Romestaing P1 Lehingue Y Carrie C Coquard R Montbarbon X Ardiet JM Mamelle N Gérard JP Role of a 10-Gy boost in the conservative treatment of early breast cancer: results of a randomized trial in Lyon, France J Clin Oncol 1997 15 963 968 9060534 10.1200/JCO.1997.15.3.963

[B31] Bartelink H Horiot JC Poortmans PM Struikmans H Van den Bogaert W Fourquet A Jager JJ Hoogenraad WJ Oei SB Wárlám-Rodenhuis CC Pierart M Collette L Impact of a higher radiation dose on local control and survival in breast-conserving therapy of early breast cancer: 10-year results of the randomized boost versus no boost EORTC 22881-10882 trial J Clin Oncol 2007 25 3259 3265 10.1200/JCO.2007.11.4991 17577015

[B32] Ceilley E Jagsi R Goldberg S Grignon L Kachnic L Powell S Taghian A Radiotherapy for invasive breast cancer in North America and Europe: results of a survey Int Radiat Oncol Biol Phys 2005 61 365 373 10.1016/j.ijrobp.2004.05.069 15667954

[B33] Freedman GM Anderson PR Goldstein LJ Ma CM Li J Swaby RF Litwin S Watkins-Bruner D Sigurdson ER Morrow M Four-week course of radiation for breast cancer using hypofractionated intensity modulated radiation therapy with an incorporated boost Int J Radiat Oncol Biol Phys 2007 68 2 347 353 10.1016/j.ijrobp.2006.12.035 17379430

[B34] Formenti SC Gidea-Addeo D Goldbergn JD Goldberg JD Roses DF Guth A Rosenstein BS DeWyngaert KJ Phase I-II trial of prone accelerated intensity modulated rdaiation therapy to the breast to optimally spare normal tissue J Clin Oncol 2007 25 16 2236 2242 10.1200/JCO.2006.09.1041 17470849

[B35] Chadha M Vongtama D Friedmann P Parris C Boolbol SK Woode R Harrison LB Comparative acute toxicity from whole breast irradiation using 3-week accelerated schedule with concomitant boost and the 6.5-week conventional schedule with sequential boost for early-stage breast cancer Clin Breast Cancer 2012 12 1 57 62 10.1016/j.clbc.2011.09.002 22056970

[B36] Deantonio L Gambaro G Beldì D Masini L Tunesi S Magnani C Krengli M Hypofractionated radiotherapy after conservative surgery for breast cancer: analysis of acute and late toxicity Radiat Oncol 2010 5 112 10.1186/1748-717X-5-112 21092288 PMC3000406

[B37] Plataniotis GA Dale RG FIPEM., FRCR. Biologically effective dose-response relationship for breast cancer treated by conservative surgery and postoperative radiotherapy Int J Radiat Oncol Biol Phys 2009 75 512 517 10.1016/j.ijrobp.2009.05.013 19625139

[B38] Corbin KS Dorn PL Jain SK Al-Hallaq HA Hasan Y Chmura SJ Hypofractionated radiotherapy does not increase acute toxicity in large-breasted women: results from a prospectively collected series Am J Clin Oncol 2013 Epub ahead of print 10.1097/COC.0b013e31827b45b7 23357972

[B39] Tortorelli G Di Murro L Barbarino R Cicchetti S di Cristino D Falco MD Fedele D Ingrosso G Janniello D Morelli P Murgia A Ponti E Terenzi S Tolu B Santoni R Standard or hypofractionated radiotherapy in the postoperative treatment of breast cancer: a retrospective analysis of acute skin toxicity and dose inhomogeneities BMC Cancer 2013 13 230 10.1186/1471-2407-13-230 23651532 PMC3660202

[B40] Chen MF Chen WC Lai CH Hung CH Liu KC Cheng YH Predictive factors of radiation-induced skin toxicity in breast cancer patients BMC Cancer 2010 10 508 10.1186/1471-2407-10-508 20860847 PMC2955039

[B41] Tsang Y Haviland J Venables K Yarnold J FAST Trial Management Group The impact of dose heterogeneity on late normal tissue complication risk after hypofractionated whole breast radiotherapy Radiother Oncol 2012 104 143 147 10.1016/j.radonc.2012.06.002 22809586

